# Reliability and minimal detectable change of dynamic temporal summation and conditioned pain modulation using a single experimental paradigm

**DOI:** 10.1371/journal.pone.0307556

**Published:** 2024-07-25

**Authors:** Matthieu Vincenot, Louis-David Beaulieu, Louis Gendron, Serge Marchand, Guillaume Léonard

**Affiliations:** 1 Research Center on Aging, CIUSSS de l’Estrie-CHUS, Sherbrooke, Québec, Canada; 2 Faculty of Medicine and Health Sciences, Université de Sherbrooke, Sherbrooke, Québec, Canada; 3 BioNR Research Lab, Université du Québec à Chicoutimi, Chicoutimi, Canada; 4 Faculty of Medicine and Health Sciences, Department of Pharmacology-Physiology, Université de Sherbrooke, Sherbrooke, Québec, Canada; 5 Research Center of the Centre Hospitalier Universitaire de Sherbrooke, Sherbrooke, Québec, Canada; 6 Faculty of Medicine and Health Sciences, Department of Surgery, Université de Sherbrooke, Sherbrooke, Québec, Canada; 7 Faculty of Medicine and Health Sciences, School of Rehabilitation, Université de Sherbrooke, Sherbrooke, Québec, Canada; University of Stavanger: Universitetet i Stavanger, NORWAY

## Abstract

**Background:**

Quantitative sensory tests (QST) are frequently used to explore alterations in somatosensory systems. Static and dynamic QST like pain threshold and temporal summation (TS) and conditioned pain modulation (CPM) are commonly used to evaluate excitatory and inhibitory mechanisms involved in pain processing. The aim of the present study was to document the reliability and the minimal detectable change (MDC) of these dynamic QST measurements using a standardized experimental paradigm.

**Material and methods:**

Forty-six (46) pain-free participants took part in 2 identical sessions to collect TS and CPM outcomes. Mechanical (pressure pain threshold [PPT]) and thermal (constant 2-minute heat pain stimulation [HPS]) nociceptive stimuli were applied as test stimuli, before and after a cold-water bath (conditioning stimulus). TS was interpreted as the change in pain perception scores during HPS. CPM were determined by calculating the difference in pain perception between pre- and post- water bath for both PPT and HPS. Relative and absolute reliability were analyzed with intra-class correlation coefficient (ICC_2, k_), standard error of the measurements (SEM_eas_) and MDC.

**Results:**

Results revealed a good to excellent relative reliability for static QST (ICC ≥ 0.73). For TS, a poor to moderate relative reliability depending on the calculation methods (ICC = 0.25 ≤ ICC ≤ 0.59), and a poor relative reliability for CPM (ICC = 0.16 ≤ ICC ≤ 0.37), both when measured with mechanical stimulation (PPT) and thermal stimulation (HPS). Absolute reliability varied from 0.73 to 7.74 for static QST, 11 to 22 points for TS and corresponded to 11.42 points and 1.56 points for thermal and mechanical-induced CPM, respectively. MDC analyses revealed that a change of 1.58 to 21.46 point for static QST, 31 to 52 points for TS and 4 to 31 points for CPM is necessary to be interpreted as a real change.

**Conclusion:**

Our approach seems well-suited to clinical use. Although our method shows equivalent relative and absolute reliability compared to other protocols, we found that the reliability of endogenous pain modulation mechanisms is vulnerable, probably due to its dynamic nature.

## Introduction

Pain is a complex phenomenon resulting from the interplay between excitatory and inhibitory mechanisms. Temporal summation (TS) refers to an excitatory pain mechanism implying a gradual enhancement of pain perception during repeated or constant painful stimulations [1,2]. Conditioned pain modulation (CPM) delineates a phenomenon where pain itself serves as an inhibitory mechanism for further pain perception. It involves a pain-inhibiting mechanism wherein a noxious stimulation results in widespread hypoalgesia [1,3,4]. Although involving distinct underlying processes, TS and CPM are often compared to sensitization [5] and diffuse noxious inhibitory control [6] respectively, two neurophysiological phenomena initially studied in animals. In the last decade, TS and CPM have been proposed as potential biomarkers to predict treatment success and guide personalized medicine [7,8]. However, evidence supporting their metrologic properties, particularly their reliability, remains uncertain [9,10].

Reliability is an umbrella term referring to the ability to give error-free measurements in stable individuals, while responsiveness refers to a measurement detecting changes, over time [11]. TS and CPM measures, like any other measurement, require both high reliability and responsiveness to be considered useful for diagnostic, prognostic, and personalized care purposes. Concerning *relative* reliability (i.e., the degree to which stable individuals in a sample maintain their position relative to each other with repeated measurements [11,12]), studies reveal indices ranging from poor to excellent for TS- and CPM-related measures [9]. Several factors have been put forward to explain these variations across studies, such as the protocol used and the methods for calculating reliability [9,10,13]. While *relative reliability* (best measured with the intraclass correlation coefficient [ICC[14]]) is useful to explore if a measurement can reliably detect differences between individuals, it is not best suited to judge the measurement’s ability to track changes, over time [12,15]. To this end, *absolute reliability* (or measurement error)–which estimates the degree of error in a person’s score in the absence of true changes [11], and is ideally measured by the standard error of the measurement [14,16] (SEMeas)–is best suited. SEMeas allows to estimate the minimal detectable change (MDC), which in turn informs on the measurement’s responsiveness [17]. Unfortunately, SEMeas and MDC have rarely been reported for TS and CPM [9], and no studies have yet documented these values for protocols using constant thermal stimulations.

The aim of this study was to fill this knowledge gap by assessing absolute and relative reliability and responsiveness of TS and CPM using a single experimental procedure. We were particularly interested in the impact of calculation methods on reliability performance.

## Materials and methods

### Participants

Forty-six (46) healthy participants were enrolled in this study and in which they participated in 2 identical testing sessions. In fact, there is no formal sample size calculation for reliability studies to ensure a minimum of significance. Although it is generally recommended to recruit between 20 and 40 participants [18], the confidence interval is another important element to consider, as it provides a minimum level of confidence in the ICC estimate. Each session lasted approximatively 1.5 hour and took place on different days, within a 1-week period. Inclusion and exclusion criteria are presented in Table 1. We asked participants to refrain from consuming tobacco [19,20], alcohol [21,22], stimulants [23] (caffeine, theine; etc.), as well as pain medication (e.g,. acetaminophen, non-steroidal anti-inflammatory drugs, and narcotics) during the 24-hour period preceding each experimental session.

**Table 1:** Inclusion and exclusion criteria.

Inclusion criteria	Age between 18 and 79 years old
	Pain free
	Able to understand French or English
Exclusion criteria	Pregnancy or post-partum (<1 year)
	Active cancer or cancer-related pain
	Cardiovascular, psychotic, or neurocognitive disorder
	Regular use of recreational substances
	Arm sensitivity loss

### Ethic approval

This cross-sectional study was conducted at the Centre de recherche du Centre hospitalier universitaire de Sherbrooke (Sherbrooke, Quebec, Canada). The recruitment occurred from February 4, 2019, to July 14, 2019, following the principles outlined in the Helsinki Declaration and the International Council for Harmonisation of Good Clinical Practice. Ethics approval was granted from the institutional review board of the Centre intégré universitaire de santé et de services sociaux de l’Estrie ‐ Centre hospitalier universitaire de Sherbrooke (CIUSSS de l’Estrie ‐ CHUS), Sherbrooke, Canada. The trial was registered on clinicaltrial.gov (NCT03376867). A written informed consent was obtained for each participant.

### Experimental setup

All sessions were conducted in the same room. For a same participant, sessions were conducted at the same time of the day to avoid circadian effect [24]. The same experimenter realized all experiments. TS and CPM were assessed within a single quantitative sensory testing experimental procedure [4,25,26]. Participants’ eligibility was confirmed, and their written informed consent was obtained before beginning the session. TS and CPM were performed based on the protocol described by Tousignant-Laflamme et al. [26]. This protocol allows for the measurement of both TS and CPM within only one dynamic quantitative sensory testing procedure, by administering test stimuli before and after a conditioning stimulus. Testing sessions were carried out in a quiet and temperature-controlled room. Experiments were conducted in a comfortable chair.

### Dynamic quantitative sensory testing

#### Equipment

A mechanical test stimulus was administered by determining the pressure pain threshold (PPT), during which mechanical pressure was gradually increased over the upper trapezius muscle until the participant reached the early first sensations of pressure pain. The mechanical test stimulus was administered via a digital algometer (*Jtech Medical*, *Midvale*, *Utah*, *USA*). A thermal test stimulus was administered using a 3 cm^2^ thermode (*TSA II*, *Thermal Sensory Analyzer*, *Medoc*, *Ramat Yishai*, *Israel*) applied to each participant’s right (calibration) and left (test stimulus) forearm. For thermal test stimulus, pain perception was continuously recorded with a computerized visual analog scale (CoVAS) which consists of a slider running along a 100 mm horizontal track, housed in a box. Participants were asked to rate their pain by moving the slider between the left boundary (identified as “no pain” ‐ score = 0) and the right boundary (identified as “maximum you can tolerate” ‐ score = 100). We also included a numerical scale above the slider to give participants reference points. The CoVAS sampling rate was set at 10 Hz (10 pain measurements per second). The conditioning stimulus consisted of a cold pressor test involving a cold-water bath set at 10°C in which participants immersed their right forearm and hand for 120 seconds.

**Fig 1:**
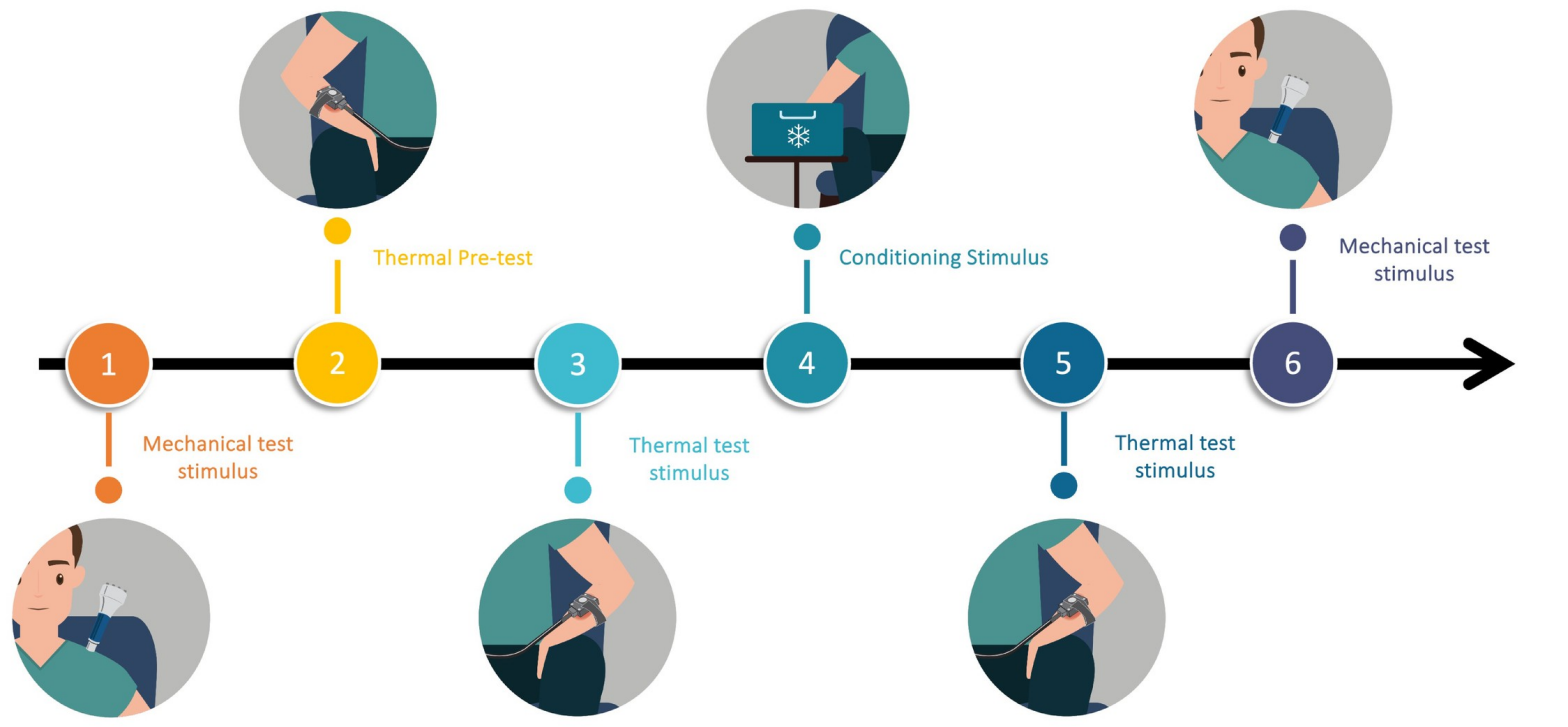
Step-by-step timeline of the procedure.

Fig 1 illustrates the step-by-step timeline of the procedure. This sequence was chosen in relation to the experts’ recommendations [4]. We favored a sequential protocol over a parallel protocol to limit possible attentional bias. We also applied 2 different types of test stimuli (mechanical and thermal), as recommended [4].

#### Pretests and test stimuli

The mechanical and thermal test stimuli were administered before and immediately after the conditioning stimulus. The mechanical test stimulus was first applied, followed by a pause (∼ 6 minutes) until the pain sensation induced by the pressure pain threshold disappeared completely. We performed a practice test before administering the thermal test stimulus to familiarize the participants with this type of stimulation. Afterwards, a pretest was performed to identify the stimulation parameters to be used as the formal heat pain test stimulus by applying the thermode on the right-side anterior forearm. The temperature was gradually increased from a baseline temperature of 32°C to a maximum of 51°C, at a rate of 0.3°C/s. The pretest consisted of 3 trials in which participants were asked for the first one to verbally identify the point at which the heat became painful (heat pain threshold ‐ HPT) and the point at which the pain was no longer tolerable (heat pain tolerance ‐ HPTol). For the 2 other trials, we asked participant to use the CoVAS to identify the HPT, HPTol and the target temperature, i.e. the temperature that induced a pain intensity rating of 50/100. We averaged the 2 last trials for the HTP, HPTol and the target temperature. The resulting target temperature was used for the formal thermal test stimulus.

The thermal test stimulus consisted of a painful thermal stimulation applied with the thermode on the left forearm, at a predetermined, individually tailored temperature (target temperature). To avoid creating expectations, participants were told that the thermode temperature could either randomly increase, remain stable, or decrease throughout the stimulation. In fact, after a constant rise (0.3°C/s) from baseline (32°C) to the predetermined temperature (pain levels of 50/100 based on pretests data), the temperature remained constant throughout the 120 seconds of the test stimulus, during which participants were asked to continuously record their pain level, using the CoVAS. The mechanical and thermal test stimuli were re-administered under the same conditions after the conditioning stimulus.

#### Conditioning stimulus

The conditioning stimulus consisted of a cold pressor test, wherein the participants immersed their right forearm and hand in a cold-water bath at 10°C for 120 s. During this cold pressor test, participants were also asked to continuously rate the intensity of their pain, using the CoVAS. These scores were used to calculate the average pain evoked by the conditioning stimulus and to ensure that a sensation of pain has been induced.

#### Temporal summation and CPM calculation

Different techniques for calculating TS and CPM have been reported in the literature. To compare techniques and determine whether one provides better reliability results, we calculated TS amplitude with 2 methods that focus on pain fluctuations prior to the conditioning stimulus over 3 different time intervals (Table 2). A positive score indicates increased pain perception and was interpreted as the presence of TS. Conversely, a negative score or a score equal to 0 was interpreted as an absence of TS. For its part, the amplitude of CPM was calculated using 7 different methods, based on the pain level fluctuation evoked by mechanical and thermal test stimuli prior to and after the conditioning stimulus. For CPM-HPS, a negative score indicates a hypoalgesic effect and a positive score or a score equal to 0 was interpreted as an absence of CPM effect. Interpretation is reversed for CPM-PPT considering the nature and characteristics of pain threshold measurement, with an increase in pain thresholds reflecting a hypoalgesic effect, and *vice versa*. Calculation methods for TS and CPM are presented in Table 2.

**Table 2:** Calculation methods for TS and CPM outcomes.

**Temporal Summation outcomes**	
TS [*interval*]	Change in pain perception during tonic thermal stimulation between the interval:
[0*-120*]	Second 0 and second 120
[30–120]	Second 30 and second 120
[60–120]	Second 60 and second 120
TS Reg [*interval*]	Change in pain perception during tonic thermal stimulation determining by the regression line between:
[0–120]	Second 0 and second 120
[30–120]	Second 30 and second 120
[60–120]	Second 60 and second 120
**Conditioned Pain Modulation outcomes**	
CPM-HPS [*interval*]	Comparison between the average pain reported during the heat pain stimulation before and after the conditioning stimulus:
[0–120]	On the interval 0–120 second
[30–120]	On the interval 30–120 second
[60–120]	On the interval 60–120 second
[110–120]	On the interval 110–120 second
[120]	At 120 second
CPM-PPT	Comparison of the mechanical pain threshold before and after the conditioning stimulus
CPM-MaxPain	Comparison between the maximum level of pain perception induced during the heat pain stimulation before and after the conditioning stimulus

* For reference: Second 0 correspond the moment when the thermode reaches the target temperature (50/100) and second 120 correspond to the end of the stimulation.

### Statistical analyses

#### Validation of assumptions

Several assumptions must be verified before performing reliability analyses [14,27]. First, the Shapiro-Wilk’s test and a visual examination of histograms and normal Q-Q plots for each outcome were considered to assess normality distributions. Second, homoscedasticity (the absence of correlation between the size of error and the magnitude of the observed score) was tested by exploring the correlation (R^2^) among the absolute difference and the mean values between the two sessions [14,28]. If R^2^ values were lower than 0.1, homoscedasticity was considered present. Systematic error between the 2 sessions was evaluated with a paired t-test, using an ***α*** threshold of .05. In cases where at least one of these assumptions (normality, homoscedasticity, absence of systematic error) was not met, the variable(s) in question was (were) not included in the reliability analyses. In fact, there is no non-parametric equivalent for ICCs, SEMeas, and MDC, and although log transformation can offer a suitable alternative for correcting non-normal or heteroscedastic distributions [29,30] it was not appropriate in the present study since our data often had negative values or values equal to 0.

#### Relative reliability

Relative reliability was determined by the ICC [16] using the ICC_2,1_ model (named 2-way random-effect “consistency” and “single measure” in SPSS [14]). Although the qualitative interpretation of ICC scores remains arbitrary [31], we applied the following benchmarks based on Koo and colleagues [32]: poor if ICC < 0.50; moderate if 0.51 ≤ ICC ≤ 0.75; good if 0.76 ≤ ICC ≤ 0.90; and excellent if ICC > 0.90. ICC estimation is coupled to the 95% confidence interval (95%CI). We also computed the coefficients of variation (CV), which represents the ratio of the standard deviation to the mean to allow for a better interpretation of ICC scores. With no real unit of measurement, it can be used to compare value distributions whose measurement scales are not comparable. The higher the coefficient of variation, the greater the level of dispersion around the mean. CV were estimated as follow: CV = (pooled mean from tests 1 & 2) / (pooled standard deviation from tests 1 & 2) *100.

#### Absolute reliability

Absolute reliability was evaluated using the SEMeas [14,28]. SEMeas were determined using the following formula: *SEMeas = $\sqrt{MSE}$* where *MSE* is the mean squared error term (or ‘‘residual error”) obtained from the ANOVA applied on test and retest measurements. For its part, the MDC was calculated from SEM scores, as follows: *MDC = 1.96*SEMeas* $\sqrt{2}$* as suggested [14,28]. 1.96 and $\sqrt{2}$ are used, respectively, to build 95% confidence intervals around SEMeas and to consider that errors are reproduced at both test and retest [14]. The MDC indicates the minimal amount of change to be reached in an individual to exceed the measurement’s error, which can then be interpreted with 95% confidence that a real change occurred. A smaller MDC indicates a more sensitive measure [33].

## Results

### Participants characteristics

All 46 participants completed the experiments and were included in the analyses. Participant’s characteristics are presented in Table 3. None of them experienced acute or chronic pain during the testing sessions, and all refrained from using pain medication for 24h hours prior to the experiments. The average interval between session 1 and 2 was 4 days (± 1.68; min: 2 days and max: 7 days).

**Table 3:** Demographic characteristics.

Age, (mean ± SD) year	47.3 ± 18
Sex ratio, n (%)	
Female	22 (52.2)
Male	24 (47.8)
Ethnicity, n (%)	
White-Caucasian	45 (97.8)
Other	1 (2.2)
Handedness, n (%)	
Right-handed	43 (93.5)
Left-handed	3 (6.5)
Language, n (%)	
French	46 (100)

### Assumption validation

All variables were homoscedastic. The variables *TS 30–120*, *TS Reg 30–120*, *CPM-HPS 0–30*, *CPM-HPS 30–120*, and *CPM-HPS 60–120* did not respect normality. Moreover, we noted a significant difference between the 2 test sessions for all CPM-HPS variables. As a result, the variables listed above were all excluded from the analyses. Group means and standard deviations for all TS and CPM outcomes and systematic differences between session 1 and 2 are presented in Table 4.

**Table 4:** Group means, standard deviation, systematic differences, homoscedasticity and normality for TS and CPM outcomes.

	Session 1		Session 2				
	*Mean*	*sd*	*Mean*	*sd*	*p*	*Normality*	*Homoscedasticity*
***Static QST***							
Heat Pain Threshold^⚬^	*38*.*27*	*4*.*53*	*38*.*16*	*4*.*14*	*ns*	*✓*	*✓*
Heat Pain Tolerance^⚬^	*47*,*76*	*1*,*84*	*47*,*80*	*1*,*67*	*ns*	*✓*	*✓*
Target Temperature^⚬^	*45*.*57*	*2*.*45*	*45*.*78*	*2*.*14*	*ns*	*✓*	*✓*
Pain during the Cold-Water Bath^⚭^	*35*.*98*	*15*.*68*	*38*.*38*	*16*.*69*	*ns*	*✓*	*✓*
Pressure Pain Threshold^⚮^	*8*.*74*	*3*.*25*	*9*.*10*	*2*.*81*	*ns*	*✓*	*✓*
***Temporal summation***							
TS [0–120]^⚭^	-3.54	29.85	-4.47	26.96	*ns*	*✓*	*✓*
TS [30–120]^⚭^	5.22	21.24	2.95	22.78	*ns*	✗	*✓*
TS [60–120]^⚭^	3.65	20.28	0.90	16.91	*ns*	*✓*	*✓*
TS Reg [0–120]^⚭^	-5.51	24.71	-4.51	27.03	*ns*	*✓*	*✓*
TS Reg [30–120]^⚭^	3.55	25.97	2.54	23.25	*ns*	✗	*✓*
TS Reg [60–120]^⚭^	2.67	19.00	0.96	18.23	*ns*	*✓*	*✓*
***Conditioned Pain Modulation***							
CPM-HPS [0–120]^⚭^	-9.68	15.87	-4.46	12.51	*0*.*02*	*✓*	*✓*
CPM-HPS [30–120]^⚭^	-10.15	17.76	-4.45	12.99	*0*.*02*	✗	*✓*
CPM-HPS [60–120]^⚭^	-10.84	19.82	-3.77	12.43	*0*.*02*	✗	*✓*
CPM-HPS [110–120]^⚭^	-9.35	21.44	-1.26	15.83	*0*.*02*	*✓*	*✓*
CPM-HPS [120]^⚭^	-9.10	21.24	-1.63	14.47	*0*.*03*	*✓*	*✓*
CPM-MaxPain^⚭^	-7.64	14.47	-4.93	14.33	*ns*	*✓*	*✓*
CPM-PPT^⚮^	1.41	1.89	0.92	1.51	*ns*	*✓*	*✓*

Units of measurement: ^⚬^: Degrees Celsius; ^⚭^: Pain level; ^⚮^: Kilogram.

### Relative reliability

ICC with 95% CI and CV are presented in Table 5. ICCs for static QST ranged from good to excellent, except for HPT, suggesting moderate reliability. ICCs for TS ranged from poor to moderate. TS amplitude calculated for the largest time interval (0–120 sec) showed the greatest ICC value. In contrast, the lowest ICC value was observed for the 60–120 sec time interval. ICCs of the TS measures calculated using the regression line were poor (0.25). ICCs for CPM were all poor, regardless of the calculation method. Still, CPM-MaxPain outcome showed a higher ICC score than CPM-MPT.

**Table 5:** Relative, absolute reliability and minimal detectable change for TS and CPM outcomes.

	ICC *[95%CI]*	CV (%)	SEMeas	MDC
***Static QST***				
Heat Pain Threshold^⚬^	0.73 *[0*.*56–0*.*84]*	11.29	1.94	5.38
Heat Pain Tolerance^⚬^	0.90 *[0*.*82–0*.*94]*	3.68	0.57	1.58
Target Temperature^⚬^	0.90 *[0*.*82–0*.*94]*	5.02	0.73	2.03
Pain during the Cold-Water Bath^⚭^	0.77 *[0*.*62–0*.*87]*	43.44	7.74	21.46
Pressure Pain Threshold^⚮^	0.76 *[0*.*60–0*.*86]*	33.91	1.49	4.13
***Temporal summation outcomes***				
TS 0–120^⚭^	0.59 *[0*.*36–0*.*75]*	-706.44	18.19	50.43
TS 60–120^⚭^	0.17 *[-0*.*12–0*.*44]*	818.98	16.97	47.04
TS Reg 0–120^⚭^	0.25 *[-0*.*04–0*.*50]*	-514.28	22.46	62.26
TS Reg 60–120^⚭^	0.25 *[-0*.*04–0*.*50]*	2177.67	11.23	31.13
***Conditioned Pain Modulation outcomes***				
CPM-MaxPain^⚭^	0.37 *[0*.*09–0*.*59]*	-228.92	11.42	31.67
CPM-PPT^⚮^	0.16 *[-0*.*13–0*.*43]*	147.38	1.56	4.37

Units of measurement: ^⚬^: Degrees Celsius; ^⚭^: Pain level; ^⚮^: Kilogram

ICC: intraclass correlation coefficient, CV: coefficient of variation, SEMeas: standard error of measurements MDC: minimal detectable change.

### Absolute reliability

SEMeas and MDCs for TS are reported in Table 5. Concerning static QST, SEMeas ranged from 0.57 to 7.74. For TS, all SEMeas values were comparable to each other, as they were expressed in the same unit. All SEMeas were under 25. The lowest SEMeas value was that associated with the smallest time interval (60–120 sec), whether calculated by subtraction or using regression analyses. On the contrary, the highest SEMeas were observed for the largest time interval (0–120 sec). As MDCs are mathematically related to the SEMeas, the largest MDC also relates to the smallest intervals, and *vice versa*. Concerning CPM, the 2 metrics are not expressed in the same unit, thus preventing direct comparisons. Nevertheless, the CPM MaxPain variable has a relatively low SEMeas, close to those observed for the TS. SEMeas and MDCs for CPM are presented in Table 5.

## Discussion

There is increasing clinical interest in TS and CPM measures, particularly due to their potential utility in identifying individuals at risk of developing chronic pain and predicting the success of pain treatments. The development of reliable measurements remains an important prerequisite to achieve this goal. The objective of this study was to evaluate the relative and absolute reliability of a series of TS and CPM measurements obtained using a standardized protocol with static and dynamic QST measures.

### Relative reliability

The results of our analyses on static QST showed that the values of the relative reliability indices are moderate to excellent, consistent with previous studies examining thermal and mechanical pain thresholds [9,24,34–37], and CPT pain [10]. Regarding the target temperature, our analyses show that this variable remains relatively stable over time and can therefore be considered as a good indicator for performing the stimulus test.

It should be reminded that the ICC results are group-dependent and are therefore difficult to generalize. ICCs allow us to determine what proportion of the observed variability of the measurement can be attributed to an error of measurement, compared with the variability between individuals in the group [14,16]. From a clinical point of view, ICC indicates the extent to which a given test is able to discriminate between different individuals, and whether their relative position in the sample is maintained when the test is reproduced [14,16]. However, very large or very small group variability (as expressed for example by group CVs) tends to respectively over- or under-estimate ICC, independently of the actual reliability of the measure [14]. The CVs observed for QST values in the present study were quite low, indicating low inter-subject variability. This, combined with their respective ICCs, confirms the good relative reliability of the static QST variables derived from our protocol. This low inter-subject variability also has the effect of lowering the ICC coefficient, possibly explaining why these coefficients were sometimes lower than those observed in other studies. As recommended [14], our ICC results should be compared and generalized to samples of healthy persons presenting with similar group variation (i.e. group CVs).

Concerning TS, few studies have attempted to document test-retest reliability, and, to the best of our knowledge, no previous study has attempted to investigate the reliability of TS induced by a 2-minute continuous HPS applied to the forearm. Our results indicate that TS induced by such a pain paradigm has poor to good relative reliability indices. Some studies have used a phasic thermal stimulation paradigm [13,38] and show poor to excellent ICCs. In this context, it has been shown that increasing the number of trials can increase ICC values [13,28]. Training most likely allows the participant to become better at succeeding at the task. This could be an interesting avenue to improve our protocol. For studies that induced TS with a mechanical phasic paradigm, the range of ICCs was wider, from poor [28] to excellent [28,39]. Strikingly, our results showed that the larger the calculation interval, the greater the ICC. Averaging over a wider interval and including more data points could potentially reduce intra-individual variability, thus increasing ICC values. Finally, we observed that smoothing the response curve with the regression line (reduce micro-variation of the VAS sampling) did not improve relative reliability. These results are coherent with the observations of Kong et al. [13]. In light of our results and in the context of measuring TS amplitude induced by continuous thermal stimulation, it appears that the 0–120 second interval would be the preferred calculation method to ensure moderate relative reliability. However, the CV value associated with this calculation interval suggests that the sample data is highly dispersed. Also, high inter-subject variability increases the likelihood of increasing the ICC.

For CPM measurement, our results show poor reliability for both thermal and mechanical test stimuli. This is coherent with the conclusion of a recent systematic review and meta-analysis [9], which revealed that reliability indices for CPM vary from poor to moderate. Using a protocol similar to ours, Kovacevic et al. [34] observed a good reliability index for CPM measures using PPT and HPS applied on the paravertebral musculature of the lumbar segment. Conversely, Naugle and al. [38], who performed PPT measurements on the forearm, obtained results much closer to ours (ICC: 0.17), suggesting that the site of stimulation could be an important factor. Complementary observations by Marcuzzi et al. [35] suggest that CPM measures tend to be more reliable when the test stimulus is based on a series a HPS, than when they are based on PPT. However, Marcuzzi et al. observed only marginally better ICCs than we did (ICC 0.50 and 0.35 for thermal and mechanical stimulation, respectively). Finally, a commendable study by Gehling et al. [40] showed that ICC were higher (increased reliability) when the stimulus test was applied during the conditioning stimulus (ICC: 0.62) compared to when it was applied 5 minutes after the conditioning stimulus (ICC: 0.17), suggesting that the timing between the second test stimulus and the conditioning stimulus can significantly affect reliability. In our study, the test stimulus was administered immediately after the conditioning stimulus. Interestingly, the ICC value in our study (ICC = 0.37) falls between these 2 values. Nevertheless, the application of the second test stimulus during the conditioning stimulus poses has certain drawbacks, notably regarding its potential impact on attention and the possibility of inducing distracting effect. Applying the second test stimulus immediately after the conditioning stimulus could represent an ideal compromise for maximizing fidelity while avoiding distraction effects [4].

Another notable point is the systematic error between the average pain perceived pre-post water bath. We noticed that the systematic error for second session was systematically lower. These observations may suggest a cognitive bias or an effect on anxiety. For instance, after completing the CPT procedure once, participants might experience less anxiety during the second session, potentially leading to reduced pain perception.

Based on our results, we recommend the use of continuous thermal stimulation over mechanical stimulation to test CPM. However, as with TS, the CVs associated with CPM ICCs are very high (in the order of 150–220%), and we must therefore be cautious in our interpretation. It should be remembered that like TS, the inter-individual variability of CPM is inherent to these mechanisms [9]. We must also keep in mind that measurement reliability of inhibitory mechanisms is dependent on methodological [9,41] and individual factors [10]. However, relying solely on the methodological argument is not enough to explain the low reliability of CPM. It is plausible that CPMs exhibit substantial variability over time, due to various biological and emotional factors. Therefore, it would be more fitting to perceive endogenous inhibitory mechanisms as dynamic processes over time, rather than stable “traits”.

### Absolute reliability

SEMeas inform us on the extent to which the results of the same measurement can vary from one observation to another, due to random factors such as measurement errors or natural variations. In practical terms, a lower SEMeas value indicates greater reliability and consistency between repeated measurements [14]. SEMeas are rarely reported in QST studies [9]. The MDC, also infrequently reported, represents the amount of change below which there is a high probability (95% chance) that no real change has occurred.

Concerning QST parameters for thermal pain perception tolerance thresholds, the SEMeas values observed in our study closely match those reported in the literature [35,36,39], demonstrating high stability over time. Unfortunately, concerning PPT, it is difficult to compare between studies, since the unit of measurement (kg, kPa, kg/cm2) and equipment (algometer, von Frey filaments) are different. Moreover, no previous studies referenced SEMeas for pain perception during the cold pressor test and the target temperature (temperature used for HPS). Our data could therefore serve as a starting point for further studies, especially as the search for the target temperature for thermal stimulation of both TS and CPM, as well as the measure of pain perception during the water bath, are relatively common in this type of protocol.

Concerning TS, SEMeas varied from 11.23 to 22.46, depending on the calculation methods. In contrast to the relative indices, the shortest measurement intervals (60–120 seconds) were associated with the best metrics (i.e., lowest SEMeas and lowest MDC). These results indicated that an individual’s TS response can be expected to vary between 11 (for TS 60–120) to 22 points (for TS 0–120) around its true score between measurements, representing the natural variation of the test. Given the absence of comparative data, it is challenging to determine the significance of this score. Yet, within our protocol, the theoretical TS range spans from -100 (complete pain perception decrease) to +100 (complete pain perception increase). In this context, SEMeas of 11 to 22 points constitute only 5.5 to 11% of the total potential score variation, which seems plausible to attain, in response to an intervention or in the presence of pain or disease.

MDC gives us another interesting piece of information. Our results indicate that a shift of 31 to 62 points on a 0–100 pain scale, contingent on the calculation method, would be essential to discern a genuine change. This is especially pertinent, for example, when implementing medication to alleviate TS in individuals grappling with persistent pain[8,42]. It would be important to consider further studies in this little-studied area, to identify parameters that influence error of measurements of TS.

Concerning CPMs, absolute reliability is rarely reported in studies [9]. Overall, the SEMeas resulting from our analyses are fairly consistent with previous studies using similar protocols with SEMeas around 10 to 15 (on a 0–100 pain scale) [35,40,41]. MDCs reported from the above-mentioned studies show the same tendency. We identified only one study reporting lower SEMeas, using the suprathreshold heat pain response as a test stimulus [43]. When CPM are calculated with PPT, our analyses reveal larger SEMeas compared with stimulation applied on the arm as opposed to the trapezius or another part of the body [35,41,44]. It would therefore seem more appropriate to apply the test stimulus to the arm.

### Strengths and limitations

This study has some limitations. The first concerns the repeated application of 2 sequential test stimuli (mechanical and thermal tests), which may have influenced measurements. However, applying PPT and HPS at distant points could have helped minimize the uncontrolled effects of CPM. Lack of randomization between mechanical and thermal modalities is a second limitation. We chose not to randomize the sequence of the 2 stimulus tests, consistently beginning instead with the shorter mechanical test to minimize the time between CPT and the second stimulus test [34]. Moreover, this study, like almost all other reliability studies, focused on short-term reliability. Future studies, looking at long-term reliability, are needed. We also wanted to emphasize that these results are specific to the protocols presented. It would be interesting for the measurement methods of TS and CPM to be standardized in terms of practice, as the DNFS has done, considering the clinical implications of these mechanisms. Finally, like any other measure of perception, it is important to remind and emphasize the subjective nature of pain measurement. Despite these limitations, our results align with previous findings. Strengths include alignment with expert group recommendations [4] and prevailing literature practices for the evaluation of TS and CPM [9,10]. Test-retest data from a larger protocol with 400+ participants would showcase experimenter expertise with a relatively large sample size compared to others [9] for better generalization.

## Conclusion

Overall, our study shows that the reliability of excitatory and inhibitory endogenous pain modulation mechanisms is quite labile and probably suffers from its dynamic nature. The method we have developed demonstrates competitive relative reliability compared with other protocols. The absolute reliability and minimal detectable change highlighted here shed light on a shadowy and clinically important point for future studies, notably on the personalization of pain medication. Further studies need to be proposed to properly compare results and identify the factors involved in these mechanisms.
